# Rivaroxaban and apixaban in patients with atrial fibrillation; a real-world data

**DOI:** 10.55730/1300-0144.5395

**Published:** 2022-03-12

**Authors:** Onur ASLAN, Sinan YILDIRIM

**Affiliations:** 1 Department of Cardiology, Tarsus State Hospital, Mersin, Turkey; 2 Department of Emergency Medicine, Çanakkale Mehmet Akif Ersoy State Hospital, Çanakkale, Turkey

**Keywords:** Rivaroxaban, apixaban, atrial fibrillation, ischemic stroke, appropriate dose

## Abstract

**Background/aim:**

This study aims to analyze the real-life data of patients who were prescribed rivaroxaban and apixaban and to emphasize the points that we think will make a difference compared to randomized controlled studies.

**Materials and methods:**

The patients who accepted to participate in the study in whom rivaroxaban (15–20 mg) and apixaban (2.5–5 mg) were started with the diagnosis of atrial fibrillation between 01 January 2018 and 31 December 2019 and whose records were fully accessed through the hospital automation system were included in the study.

**Results:**

One hundred and ninety-four (48.5%) of a total of 400 patients using rivaroxaban and apixaban were women. The mean age was 73.34 ± 10.45 years, and the age range was 41–98. There was no significant difference in terms of demographic characteristics, background information of the patients, and the medications. Drug-induced complications and mortality rates were also similar. The GFR change rates of the patients in both groups were similar even though the initial GFRs were significantly higher in rivaroxaban group. The mean age and ejection fractions of the patients using rivaroxaban 15 mg were found to be lower than those of patients using rivaroxaban 20 mg whereas the mean systolic blood pressure and HAS-BLED score were found to be higher. Ischemic stroke and mortality rates were higher in patients using 15 mg rivaroxaban than patients using 20 mg rivaroxaban. The rates of nonmajor bleeding in patients using rivaroxaban 15 mg were lower compared to those using 20 mg, and this difference was statistically significant.

**Conclusion:**

Stroke rates were found to be higher and to have similar bleeding rates compared to major clinical studies in our real-life analysis. However, high ischemic cerebrovascular event and low nonmajor bleeding rates are remarkable in low dose use of rivaroxaban. It is clear that there is a need to consider existing dose reduction criteria in terms of correct prescribing.

## 1. Introduction

Atrial fibrillation (AF) continues to be the most commonly diagnosed rhythm disorder worldwide and the number of diagnoses for the disorder has been increasing rapidly. Thus, the prevalence of pathologies associated with AF increases with age, the prevalence of AF increases in parallel to this situation [1]. Stroke is one of the severe consequences of AF [2]. Prevention of stroke and systemic thromboembolism continues to be the cornerstone of AF treatment. A treatment administered for this purpose, oral anticoagulation may prevent most stroke cases stemming from AF [3,4]. Vitamin K antagonists (VKA) and new generation oral anticoagulant (NOAC) agents are used in this regard. NOACs have been compared to warfarin in terms of efficiency and safety and they have been approved in the treatment of AF in the studies conducted. These studies have shown that NOAC group medications are at least as effective as VKA and safer at some points [5–8]. Following these studies, real-life data began to be published and the efficacy and safety results were compared according to major studies, regional prescribing habits, and physicians’ approaches were evaluated. Less selected cohorts in real-life studies can help us understand the impact of NOACs in some specific scenarios and situations compared to clinical studies [9]. However, anticoagulant therapies are long-term treatments that do not target existing symptoms; medication adherence is significantly lower in observational studies compared to clinical studies [10–12].

Our aim in this study is to evaluate the real-life data of the patients using rivaroxaban and apixaban, by utilizing activity and safety parameters. Furthermore, the study aims to determine certain parameters which can be crucial in clinical practice, such as the properties of patient profiles, prescription habits, how the dose reduction criteria are evaluated, and to identify the similarities and differences between these parameters and those of major studies.

## 2. Materials and methods

This study was conducted in a state hospital, where 150 patients on average daily and 3000 patients applied annually. Patients who were diagnosed with AF and started treatment were included in the study among the patients who applied to the hospital between 01 January 2018 and 31 December 2019. The patients included in the study were selected retrospectively after the medication reports were issued in order not to affect the treatment algorithm of the physicians in this process. The patients in whom rivaroxaban (15–20 mg) and apixaban (2.5–5 mg) treatment started were contacted and detailed information was given about the study. Patients were followed up prospectively for 1 year. Consent forms were signed by the patients who were eligible for the study after 1 year.

The patients (n: 400) who accepted to participate in the study in which rivaroxaban (15–20 mg) and apixaban (2.5–5 mg) were used after the diagnosis of AF, and whose records were fully accessible through the hospital automation system were included in the study. Patients who refused to participate in the study (n: 146), whose medications were changed for any reason (n: 38), who changed their city of residence or hospital during their follow-up period (n: 92), whose data were not fully accessible through the hospital automation system (n: 19), and who were excluded from the study due to other reasons (n: 5) were excluded from the study (Table 1).

Patient data were obtained from the hospital automation system and patient files and the data were recorded in a previously prepared form. All emergency and hospital applications of the patients were questioned during the 1-year follow-up period. The approvals of the patients were obtained and their suitability for the study was evaluated with a second format at the end of the follow-up period.

Rivaroxaban (15–20 mg) and apixaban (2.5–5 mg) were started in 720 of 2900 patients with AF in a 2-year period, and the data of 400 patients who completed the study and met the inclusion criteria were analyzed.

The mortality information of the patients was confirmed from hospital records and the death reporting system. A medication tracking system in pharmacies was used to verify the accuracy of the medications patients used. The evaluation of major bleeding was made in accordance with Internal Society on Thrombosis and Hemostasis (ISTH) major bleeding criteria [13]. Any bleeding that the clinician was aware of was considered nonmajor bleeding.

Atrial fibrillation was diagnosed according to European Society of Cardiology (ESC) guidelines for the diagnosis and management of atrial fibrillation developed in collaboration with the European Association of Cardio-Thoracic Surgery (EACTS).

Transient ischemic attack (TIA) and ischemic cerebrovascular event (CVE) were described according to the American Heart Association/American Stroke Association (AHA/ASA) 2019 Updated Guidelines; TIA is defined as an acute focal cerebral or ocular loss of function whose symptom lasts shorter than 24 h and which is thought to have been due to embolic or thrombotic vascular disease after sufficient examination.

Patients who have acute restricted-diffusion in magnetic resonance imaging (MRI), in addition to the neurological symptoms, are considered to have ischemic CVE.

Modification of diet in renal disease (MDRD) Calculation in glomerular filtration rate (GFR) calculation: GFR=175 × ([Serum creatinine]^−1.154^) × ([Age]^−0.203^) × (0.742 if female) × (1.212 if black) was used.

Approval of local authority for this study had been taken with the official paper no. E-66442466-604.01.01.

### 2.1. Statistical analysis

The data were recorded in the SPSS 17.0 package software. Among the continuous variables, those with normal distribution were expressed as mean ± standard deviation, and those without normal distribution were expressed as the median (minimum–maximum); categorical variables were expressed with numbers and percentages. The Mann–Whitney U test was used in groups without normal distribution, and Student’s t-test was used in groups with normal distribution for the significance of the difference between the means of the groups in continuous variables. Pearson’s chi-squared test and, where appropriate, Fisher’s exact test were used in order to test the significance of the difference between categorical variables.

p-values less than 0.05 were considered statistically significant.

## 3. Results

The number of women in our study was 194 (48.5%) of a total of 400 patients using rivaroxaban and apixaban. The mean age was 73.34 ± 10.45, and the age range was 41–98. Of 201 patients using rivaroxaban, 44 (21.9%) used 15 mg form, 157 (78.1%) used 20 mg form, and of 199 patients using apixaban, 24 (12.1%) used 2.5 mg form, 175 (87.9%) used 5 mg form. There was no significant difference in terms of demographic characteristics, background information of the patients, and the medications they used, when the patients using rivaroxaban and apixaban were compared. Medication-induced complications and mortality rates were also similar. The CHA_2_DS_2_–VASc and HAS-BLED scores were higher in the group in which apixaban was started but this difference was not statistically significant. Although the rates of ischemic stroke and TIA were lower in the rivaroxaban group compared to the apixaban group, this difference also was not statistically significant (p: 0.253). Major (p: 0.126) and nonmajor (p: 0.183) bleeding rates were lower in the rivaroxaban group compared to the apixaban group but there was no statistical significance. The difference between all-cause mortality rates was also statistically insignificant (p: 0.644). The GFR change rates of the patients in both groups were similar, even though the initial GFRs were significantly higher in the group in which rivaroxaban was started (p: 0.015) (Table 2).

The mean age (p: 0.002) and ejection fractions (EF) (p: 0.002) of the patients using 15 mg were found to be lower (p: 0.008), whereas the mean systolic blood pressure and HAS-BLED score were found to be higher (p: 0.005) considering the comparison between the patients who used rivaroxaban 15 mg and 20 mg. Ischemic stroke (p: 0.005) and mortality rates (p: 0.043) were higher in patients using 15 mg rivaroxaban compared to patients using 20 mg rivaroxaban (Table 3). The rates of nonmajor bleeding in patients using rivaroxaban 15 mg were lower compared to those using 20 mg and this difference was statistically significant (p: 0.047).

The mean age of the patients using 2.5 mg was higher considering the comparison between the patients using 2.5 mg and 5 mg apixaban (p: 0.001). The background characteristics, medications, complications, and mortality rates of both groups were similar (Table 4).

Low-dose rivaroxaban was started only in 28.6% (n: 8) of the patients (n: 28) with a GFR of 15–50 mL/min when evaluated according to the GFR, which is a dose-reduction indication for rivaroxaban. Seventy-one point four percent of patients with a GFR of 15–50 mL/min were started on an inappropriately standard dose of rivaroxaban. Of the patients (n: 15) who met two of the dose-reduction criteria for apixaban (creatinine > 1.5, over 80 years old, below 60 kg), 33.3% (n: 6) were started with a low dose and 66.7% (n: 9) were started with a high dose. Among patients using 20 mg of rivaroxaban, 10.83% (n: 17) of the patients met dose reduction criteria. When the same analysis was performed for apixaban, up to 7.43% (n: 13) of the patients using 5 mg met dose-reduction criteria.

Dose-reduction criteria were established during the follow-up in 3.18% (n: 5) of the patients when the patients using rivaroxaban 20 mg were evaluated, but their doses were not reduced. On the other hand, it was observed for apixaban that dose-reduction criteria were established in the follow-up of up to 1.14% (n: 2) of the patients who used 5 mg, but no dose reduction was made in these patients.

The mean age (p < 0.001), CHA_2_DS_2_–VASc (p: 0.002) and HAS-BLED (p: 0.006) scores of the patients who died were higher when the factors that may affect mortality were examined. The mean ejection fraction was statistically significantly lower in the deceased group (p < 0.001) (Table 5).

**Figure 1:**
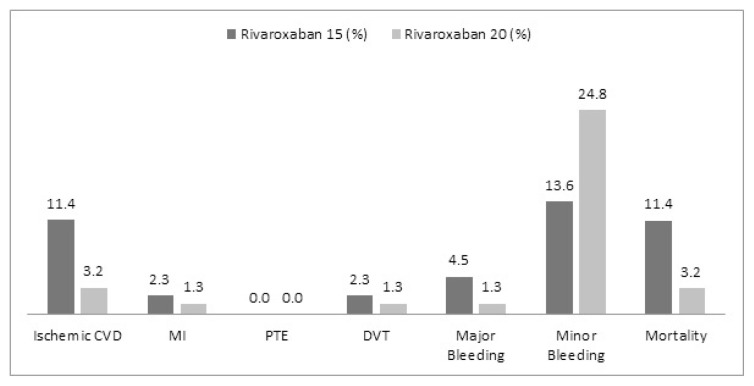
Primary efficiency-safety end points and all-cause mortality rates for rivaroxaban.

**Figure 2:**
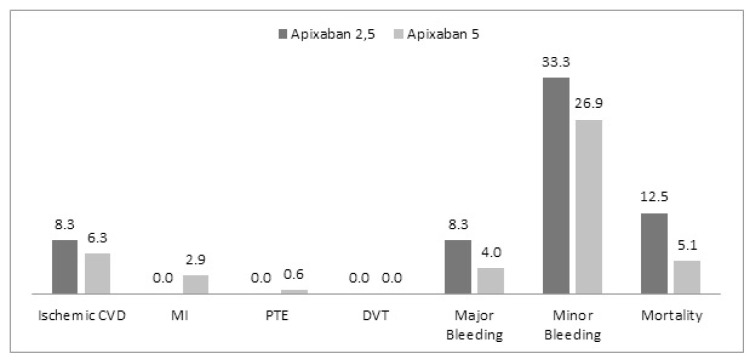
Primary efficiency-safety end points and all-cause mortality rates for apixaban.

**Figure 3:**
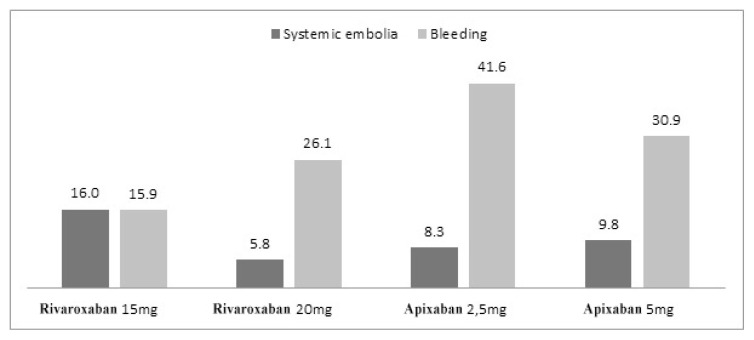
Systemic embolia and major + minor bleeding rates.

Complications and mortality numbers among patients using rivaroxaban and apixaban are presented in Figures 1 and 2, and primary endpoints for both groups are presented in detail in Figure 3.

## 4. Discussion

The major finding of the study is remarkably high rates of ischemic stroke and TIA in our real-life analysis. Secondly, even though both the bleeding rates and stroke rates tended to be higher in patients using apixaban compared to the rivaroxaban group, it was not statistically significant. However, compared with rivaroxaban 20 mg stroke rates at 15 mg of rivaroxaban were strikingly high, and nonmajor bleeding rates were again remarkably low.

Of the primary efficacy endpoints of major clinical trials; ischemic stroke and TIA, which are the most important treatment targets in patients with AF, were found to be 5% (n: 10) in the rivaroxaban group and 6.5% (n: 13) in the apixaban group. It was observed to be 3.2% (n: 5) in the 20 mg group and 11.4% (n: 5) in the 15 mg group considering the doses separately. It was determined to be 6.3% (n: 11) in the apixaban 5 mg group and 8.3% (n: 2) in the apixaban 2.5 mg group considering the doses separately. These rates are remarkably higher compared to the major clinical trials. At this point, these figures were determined as 2.1% for the intention to treat the population under the heading of stroke and systemic embolism in the ROCKET-AF study (Rivaroxaban Once Daily Oral Direct Factor Xa Inhibition Compared With Vitamin K Antagonism for Prevention of Stroke and Embolism Trial in AF), in which rivaroxaban was compared to warfarin [14]. It was reported as 0.97% under the title of ischemic or undetermined stroke in patients using apixaban in the ARISTOTLE study (Apixaban versus Warfarin in Patients with AF), in which apixaban was compared to warfarin [15]. Stroke and TIA were at the level of 0.9% in total (16) in the XANTUS study (a real-world, prospective, observational study of patients treated with rivaroxaban for stroke prevention in atrial fibrillation), in which real-life data of rivaroxaban were analyzed. At this point, stroke rates were significantly higher in all groups in our study compared to major clinical studies. The reason for this may be patients have less medication adherence in real life and clinicians tend to decide on dose reduction based on the frailty aspect of the patient rather than the criteria for dose reduction. The fact that the patient risk profile in major studies was higher than in real life may also have played a role. Ischemic stroke data of patients using low-dose rivaroxaban are particularly striking.

The use of low dose apixaban was 12%, while the rate of patients using low dose rivaroxaban was 21.8% in our study. Low dose usage rates for ROCKET-AF and XANTUS studies are 20.7% and 20.8%, respectively [14,16]. This figure was given as 4.7% in the ARISTOTLE study (15). The choice of low dose seems to be decided based on the patient’s clinical evaluation. However, the lower dose rate is higher in patients, such as older and with lower EF, who may be considered more fragile by the clinician. Only 28.6% of the patients with a GFR between 15 and 50 started low-dose rivaroxaban. Of the patients who had two or more of the dose reduction criteria for apixaban (creatinine >1.5, over 80 years old, under 60 kg), 33.3% of the patients started on low doses. It was observed that up to 10.83% of the patients had dose-reduction criteria when the patients using rivaroxaban 20 mg were evaluated, and up to 7.43% of the patients using 5 mg had dose-reduction criteria when the same analysis was performed for apixaban, but high dose medication was started instead of low dose in these patients. In the XANTUS trial, 36% of the patients were using rivaroxaban 20 mg even though there was a dose reduction indication, whereas 15% of the patients with a GFR >50 ml/min used rivaroxaban 15 mg even though they had no dose reduction criterion in the XANTUS study (16). The rate of low-dose apixaban use was reported to be 30.4% in another study evaluating the use of NOAC [17]. Nine hundred and forty-three patients were evaluated in another study in which the use of inappropriate NOAC dose was investigated and it was concluded that the low dose use rate was 13.6%, and that 70.3% of patients who take low doses were taking inappropriate doses. The rate of inappropriate high dose use was found to be 3.7% in the same study. Low dose use rates in real-life data show significant differences. This difference is also apparent in major clinical trials of apixaban and rivaroxaban. It is clear in light of these data that current standards regarding dose reduction criteria need to be further considered.

When evaluated in line with the primary safety endpoint of major clinical trials, which are major and nonmajor bleeding, the rates of bleeding in our study have been %2 (n: 4) and 4.5% (n: 9) for rivaroxaban and apixaban, respectively. The rates were 1.3% for rivaroxaban (2) 20 mg and 4.5% (n: 2) for rivaroxaban 15 mg when we evaluated the doses separately. The rate of major bleeding in patients using apixaban according to the doses was 4% (n: 7) for 5 mg, whereas it was 8.3% (n: 2) for 2.5 mg in our study. Observing more major bleeding at lower doses may have been due to the fact that low-dose patients had more comorbidities and they were older patients. A similar relationship is also present in the XANTUS study results [16]. It was stated in the ROCKET-AF study as 20.7% for rivaroxaban under the heading of major or clinically related nonmajor bleeding [14]. This rate is 5.6% when evaluated as any major bleeding. Major bleeding was observed in 1.9% of patients when evaluated alone in the XANTUS study. One of the most serious bleedings, intracranial bleeding was detected in 1 patient (n: 4) in the rivaroxaban group and in 1 patient (n: 9) in the apixaban group in our study. It was reported as 0.8% in patients with ROCKET-AF and was found to be significantly lower than warfarin [14]. Intracranial bleeding was detected as 0.4% in the XANTUS study. Major bleeding was detected as 3.8% in the ARISTOTLE study (15). It was reported to be 0.5% when intracranial bleeding rates were considered.

Another parameter that may have an important place in clinical practice and which may be significant in terms of real-life data is the other bleeding that does not meet the major bleeding criteria. There are studies showing that these bleedings have predictive value for major bleeding [18,19]. This condition, which we named nonmajor bleeding in our study, was 21.9% for the rivaroxaban group and 27.6% for apixaban. Nonmajor bleeding occurred in 13.6% (n: 6) of patients with rivaroxaban 15 mg and 24.2% (n: 38) in the 20 mg group. It was observed in 16.7% of patients with the heading of nonmajor clinical-related bleeding in the ROCKET AF study. The bleeding rate named nonmajor was given as 12.9% in patients using rivaroxaban in the XANTUS study. Nonmajor bleeding appears to be 12.1% in the ARISTOTLE study [20].

The patient population in our study had different rates in certain parameters such as age, gender, AF type, and the rate of patients with low EF, compared to major studies. Similar figures were found in terms of diabetes, hypertension, glomerular filtration rate, medications used, and CHA_2_DS_2_–VAScand HAS-BLED scores [14,15]. Previous CVD and TIA history, which may be important parameters, was 12.9% for the rivaroxaban group and 11.6% for the apixaban group in our patient population. While the same rate was strikingly 54.9% in the ROCKET-AF study, it was 19% in the ARISTOTLE study [21].

All-cause mortality rates were 5% in the rivaroxaban group and 6% in the apixaban group in our study. It was 1.9%/year in the ROCKET-AF study and 3.52%/year in the ARISTOTLE study. It was observed in our analysis that the mean age was significantly higher and EF was significantly lower in the patients who died. Comorbidities appear to be the main determinant of all-cause mortality, as supported by other studies.

There was no difference between the two groups in terms of glomerular filtration rate. There was no difference between the two groups again, and the rate of change was similar when the change in glomerular filtration rates was examined at the end of the study. Rivaroxaban and apixaban were evaluated to be safer than warfarin in terms of worsening of renal functions in a study comparing warfarin in this regard [22]. The results in our analyses also suggest that the use of rivaroxaban and apixaban does not have a clear negative effect on renal function.

## 5. Conclusion

The first treatment goal of anticoagulation in AF patients cerebrovascular with the disease, principally ischemic stroke and TIA rates were found to be higher compared with major clinical trials. Another important component of these treatments, bleeding rates were similar to major trials in our real-life analysis. Both efficacy and safety parameters tended to be worse with apixaban but there was no statistical significance. However, high ischemic CVD and low nonmajor bleeding rates are remarkable in low-dose use of rivaroxaban. More comprehensive studies are needed on this subject. Considering the dose reduction rates in the conducted studies, it is seen that the driving factor for the dose reductions is clinicians’ evaluations, rather than the standard criteria. We believe that the existing criteria should be used more effectively in terms of correct prescription to obtain maximum benefit from the medication used.

## 6. Limitations

The most important limitation of this study is the single-center design. Follow-up time may be longer than 1 year for larger-scale further studies.

## Figures and Tables

**Table 1:** Inclusion and exclusion to study randomization.

Patients with AF (n: 700) who were started with rivaroxaban and apixaban between 01.01.2018 and 31.12.2019	
Patients who used rivaroxaban (15 mg and 20 mg) (n: 358)	Patients who used apixaban (2.5 mg and 5 mg) (n: 342)
Patients who refused to participate in the study (n: 86)	Patients who refused to participate in the study (n: 60)
Patients who underwent a change of medication for any reason (n: 21)	Patients who underwent a change of medication for any reason (n: 17)
Patients who changed their city of residence or hospital during their follow-up period (n: 38)	Patients who changed their city of residence or hospital during their follow-up period (n: 54)
Patients whose data were not fully accessible through the hospital automation system (n: 8)	Patients whose data were not fully accessible through the hospital automation system (n: 11)
Patients who were excluded from the study due to other reasons (n: 4)	Patients who were excluded from the study due to other reasons (n: 1)
Rivaroxaban (15 mg and 20 mg) group (n: 201)	Apixaban (2.5 mg and 5 mg) group (n: 199)

**Table 2:** Demographic and clinical features.

		Rivaroxaban (n: 201)	Apixaban (n: 199)	p-value*
Age		72.69 ± 10.10	74.01 ± 9.85	0.292
Gender	Men	106 (52.7%)	100(50.3%)	0.619
	Women	95(47.3%)	99(49.7%)	
Weight		69.74 ± 8.41	68.31 ± 9.04	0.118
SBP		120.95 ± 14.79	122.19 ± 14.08	0.415
AF type	Paroxismal	8(4.0%)	11(5.5%)	0.467
	Chronic	193(96.0%)	188(94.5%)	
Application scores	CHA_2_DS_2_–VASc	3.40 ± 1.45	3.57 ± 1.39	0.346
	HAS-BLED	1.89 ± 0.74	1.97 ± 0.75	0.317
EF		50.27 ± 9.53	49.40 ± 9.58	0.194
Background	HT	176(87.6%)	181(91.0%)	0.273
	DM	42(20.9%)	47(23.6%)	0.513
	SVO	26(12.9%)	23(11.6%)	0.674
	MI	31(15.4%)	44(22.1%)	0.087
	PAH	5(2.5%)	2(1.0%)	0.229
	Alcohol	2(1.0%)	0	0.252
	Bleeding history	1(0.5%)	4(2.0%)	0.183
Medications used	Beta blocker	172(85.6%)	176(88.4%)	0.393
	CA channel blocker	60(29.9%)	66(33.2%)	0.475
	ACEARB	174(86.6%)	174(87.4%)	0.796
	Digoxin	85(42.3%)	98(49.2%)	0.163
	Statin	70(34.8%)	79(39.7%)	0.314
	NSAID	53(26.4%)	39(19.6%)	0.108
	PPI	151(75.1%)	135(67.8%)	0.107
	Amiodarone	3(1.5%)	3(1.5%)	0.653
	Gastric medicines	38(18.9%)	32(16.1%)	0.457
	ASA	4(2.0%)	6(3.0%)	0.369
	Klopidogrel	2(1.0%)	2(1.0%)	0.685
TFT abnormality		17(8.5%)	13(6.5%)	0.465
Initial GFR		93.80 ± 38.93	83.73 ± 29.12	0.015
GFR change		1.03 ± 24.16	0.16 ± 25.04	0.889
Complications	Ischemic CVD	10(5.0%)	13(6.5%)	0.253
	MI	3(1.5%)	5(2.5%)	0.356
	PTE	0	1(0.5%)	0.498
	DVT	3(1.5%)	0	0.126
	Major bleeding	4(2.0%)	9(4.5%)	0.126
	Minor bleeding	44(21.9%)	55(27.6%)	0.183
Mortality		10(5.0%)	12(6.0%)	0.644
Complications**	Ischemic CVD	8(4.5%)	11(6.2%)	0.487
	MI	3(1.7%)	4(2.2%)	0.503
	PTE	0	1(0.6%)	0.501
	DVT	2(1.1%)	0	0.248
	Major bleeding	4(2.3%)	8(4.5%)	0.192
	Minor bleeding	36(20.3%)	48(27.0%)	0.142
Mortality		9(5.1%)	12(6.7%)	0.508

* Mann–Whitney-U test, Pearson’s chi-squared test, and Fisher’s exact test

** Statistical analysis after excluding patients using inappropriate doses. Patients using appropriate doses of rivaroxaban (n: 177) and apixaban (n: 178).

**Table 3:** Demographic and clinical features according to doses of rivaroxaban.

		Rivaroxaban 15 (n: 44)	Rivaroxaban 20 (n: 157)	P-value*
Age		77.32 ± 8.97	71.39 ± 11.19	0.002
Gender	Men	23 (52.3%)	72 (45.9%)	0.451
	Women	21 (47.7%)	85(54.1%)	
Weight		68.52 ± 9.03	70.08 ± 8.23	0.083
SBP		116.14 ± 16.03	122.29 ± 14.18	0.008
AF type	Paroxysmal	0	8 (5.1%)	0.133
	Chronic	44 (100%)	149 (94.9%)	
Application scores	CHA2DS2–VASc	3.70 ± 1.34	3.31 ± 1.47	0.143
	HAS-BLED	2.15 ± 0.57	1.81 ± 0.77	0.005
EF		46.59 ± 10.10	51.31 ± 9.13	0.002
Background	HT	42(95.5%)	134 (85.4%)	0.054
	DM	5(11.4%)	37 (23.6%)	0.056
	SVO	8(18.2%)	18(11.5%)	0.241
	MI	4(9.1%)	27(172%)	0.139
	PAH	0	5 (3.2%)	0.287
	Alcohol	0	2 (1.3%)	0.609
	Bleeding history	0	1 (0.6%)	0.781
Medications used	Beta blocker	42(95.5%)	130(82.8%)	0.023
	CA channel blocker	8 (18.2%)	52(33.1%)	0.056
	ACEARB	41 (93.2%)	133(84.7%)	0.11
	Digoxin	15 (34.1%)	70(44.6%)	0.213
	Statin	44(100%)	157(100%)	0.636
	NSAID	10 (22.7%)	43(27.4%)	0.535
	PPI	33 (75.0%)	118(75.2%)	0.983
	Amiodarone	0	3(1.9%)	0.475
	Gastric medicines	12 (27.3%)	26(16.6%)	0.109
	ASA	0	4(2.5%)	0.369
	Klopidogrel	0	2(1.3%)	0.609
Complications	Ischemic CVD	5(11.4%)	5 (3.2%)	0.005
	MI	1 (2.3%)	2(1.3%)	0.525
	PTE	0	0	
	DVT	1 (2.3%)	2(1.3%)	0.525
	Major bleeding	2 (4.5%)	2(1.3%)	0.209
	Minor bleeding	5(11.4%)	39 (24.8%)	0.047
Mortality		5(11.4%)	5(3.2%)	0.043

* Mann–Whitney U test, Pearson’s chi-squared test, and Fisher’s exact test

**Table 4:** Demographic and clinical features and results according to doses of apixaban.

		Apixaban 2.5 (n: 24)	Apixaban 5 (n: 175)	p-value*
Age		80.21 ± 7.49	73.15 ± 9.85	0.001
Gender	Men	11(85.8%)	88 (50.3%)	0.682
	Women	13(54.2%)	87 (49.7%)	
Weight		67.46 ± 13.04	68.43 ± 8.40	0.395
SBP		120.83 ± 14.12	122.37 ± 14.11	0.578
AF type	Paroxysmal	0	11 (6.3%)	0.234
	Chronic	24(100%)	164(93.7%)	
Application scores	CHA_2_DS_2_–VASc	3.79 ± 1.14	3.54 ± 1.42	0.176
	HAS-BLED	2.08 ± 0.50	1.99 ± 0.78	0.443
EF		48.33 ± 9.63	49.54 ± 9.59	0.368
Background	HT	23(95.8%)	162(92.6%)	0.476
	DM	4(16.7%)	43(24.6%)	0.283
	SVO	3(12.5%)	20(11.4%)	0.547
	MI	7(29.2%)	37(21.1%)	0.374
	PAH	0	2(1.1%)	0.773
	Alcohol	0	0	
	Bleeding history	0	4(2.3%)	0.615
Medications used	Beta blocker	19(79.2%)	157(89.7%)	0.123
	CA channel blocker	5(20.8%)	61(34.9%)	0.126
	ACEARB	19(79.2%)	155(88.6%)	0.163
	Digoxin	12(50.0%)	86(49.1%)	0.937
	Statin	6(25.0%)	73(41.7%)	0.117
	NSAID	2(8.3%)	37(21.1%)	0.108
	PPI	15(62.5%)	120(68.6%)	0.55
	Amiodarone	1(4.2%)	2(1.1%)	0.321
	Gastric medicines	1(4.2%)	31(17.7%)	0.07
	ASA	0	6(3.4%)	0.458
	Klopidogrel	0	2(1.1%)	0.773
Complications	Ischemic CVD	2 (8.3%)	11 (6.3%)	0.615
	MI	0	5(2.9%)	0.522
	PTE	0	1(0.6%)	0.879
	DVT	0	0	
	Major bleeding	2 (8.3%)	7(4.0%)	0.297
	Minor bleeding	8(33.3%)	47(26.9%)	0.506
Mortality		3(12.5%)	9(5.1%)	0.163

* Mann–Whitney U test, Pearson’s chi-squared test, and Fisher’s exact test

**Table 5:** Demographic and clinical features of the deceased patients.

	Deceased (n: 22)	ALIVE (n: 378)	p-value*
Rivaroxaban	10 (45.5%)	191 (50.5%)	0.644
Apixaban	12(54.5%)	187 (49.5%)	
Age	80.73 ± 7.01	72.91 ± 10.47	<0.001
Women	11 (50.0%)	195 (51.6%)	0.885
Men	11(50.0%)	183 (48.4%)	
CHA_2_DS_2_–VASc	4.41 ± 1.22	3.43 ± 1.41	0.002
HAS-BLED	2.36 ± 0.73	1.90 ± 0.74	0.006
EF	41.82 ± 10.53	50.30 ± 9.30	<0.001

* Mann–Whitney U test, Pearson’s chi-squared test, and Fisher’s exact test
